# Molecular diagnosis of *Trichomonas vaginalis* in liquid-based Papanicolaou samples in Shiraz, southern Iran

**DOI:** 10.1186/s12905-022-02141-x

**Published:** 2023-01-05

**Authors:** Mohammad Saleh Bahreini, Samaneh Sedghi, Yalda Badalzadeh, Mohammad Hossein Motazedian, Manouchehr Shirani, Sareh Sami Jahromi, Aref Teimouri, Mahmoud Agholi, Qasem Asgari

**Affiliations:** 1 Department of Parasitology and Mycology, School of Medicine, Shiraz University of Medical Sciences, Shiraz, Iran; 2 Department of Pathology, School of Medicine, Shiraz University of Medical Sciences, Shiraz, Iran; 3 Department of Parasitology and Mycology, School of Medicine, Fasa University of Medical Sciences, Fasa, Iran

**Keywords:** Trichomoniasis, Papanicolaou, Iran, PCR, Liquid-based

## Abstract

**Background:**

Trichomoniasis is a parasitic infection of the urinary and genital tract, caused by *Trichomonas vaginalis*. This study aimed to investigate the molecular diagnosis of *T. vaginalis* infection in liquid-based Papanicolaou samples in Shiraz, southern Iran.

**Materials and methods:**

In this cross-sectional study, 534 liquid-based Papanicolaou samples were collected from women referring to the laboratory of Motahari Clinic of Shiraz University of Medical Sciences in 2021. Genomic DNA were extracted from the samples and examined for evidence of *T. vaginalis* using polymerase chain reaction (PCR) using TVK3 and TVK7 specific primers.

**Results:**

The mean age of participants was 39.28 ± 9.89 with a maximum age of 65 and a minimum age of 19 years. *T. vaginalis* DNA fragments were detected in 4.86% (26/534) of the cases. There was significantly higher prevalence in the age groups of 21 to 30 and 41 to 50 years (46.15%, *p* = 0.001 and 38.46%, *p* = 0.015, respectively). Furthermore, the results showed an association between a history of foamy discharge and *Trichomonas* positivity (*p* = 0.001).

**Conclusion:**

*T. vaginalis* infection is common in liquid-based Papanicolaou samples of women who attended regular health check-ups in the study area. Screening for trichomoniasis in populations, particularly if using highly sensitive methods such as PCR, may lead to increased detection and treatment.

## Background

Trichomoniasis is caused by the protozoan *Trichomonas vaginalis*, which is one of the most common sexually transmitted diseases (STD), with an estimated annual incidence of 276.4 million cases globally [1, 2]. In women, the cervix, vagina, Bartholin’s glands, bladder, urethra, and sometimes the upper genital/urinary tract are areas where *T. vaginalis* may cause infection. A frothy, yellow-green vaginal discharge with a strong, foul odor, soreness, itching, and irritation of the genital area, urination, or sexual with painful are the most important symptoms in women [3]. *T. vaginalis* adheres to vaginal epithelial cells and damages the cells and urethral tissue and more than doubles the risk of acquiring human immunodeficiency virus (HIV) [4]. Furthermore, complications of trichomoniasis include prostatitis, epididymitis, urethral stricture, and pelvic inflammatory disease in men [5]. The prevalence of *T. vaginalis* is related to several factors including past or present infection with other STDs, multiple sexual partners, and unsafe sex practices [6]. The prevalence rate of *T. vaginalis* infection has been reported differently in various regions and populations of Iran [7]. While, 10 to 50% of infections seem to be asymptomatic and undiagnosed [8]. Due to some risk factors such as lack of regular screening, lack of timely treatment, and chronic disease and drug resistance, there is a possibility of recurrent trichomoniasis in women. [9]. Wet-mount test, Diamond’s medium culture, and antigen test are the common methods of trichomoniasis diagnosis based on vaginal secretions. However, these techniques have low sensitivity in detecting low parasite loads [10–12]. Although Papanicolaou (Pap) test is one of the routine methods for the detection of potentially precancerous and malignant cells in the cervix, *T. vaginalis* is also detected in some cases. However, the method is ineffective in diagnosing *T. vaginalis* in asymptomatic women due to its low sensitivity and specificity [13, 14]. Studies have shown that molecular techniques such as polymerase chain reaction (PCR) have been widely employed for the specific detection of *T. vaginalis* [15]. However, limited information is available concerning PCR-based direct detection of *T. vaginalis* infections using liquid-based Pap samples. To the best of the authors’ knowledge, no study has been performed to detect *T. vaginalis* DNA in residual liquid-based Pap samples in Shiraz, southern Iran. Therefore, the current study was conducted to detect *T. vaginalis* DNA in liquid-based Pap samples using PCR assay.

## Materials and methods

### Ethical statement

This study was approved by the Ethics Committee of Shiraz University of Medical Sciences with an ethical code: IR.SUMS.MED.REC.1400.615. Written informed consent was obtained from all patients.

### Study design and participants

This cross-sectional study was performed on liquid-based Papanicolaou samples belonging to women who had been referred to the laboratory of Shahid Motahari Clinic affiliated with Shiraz University of Medical Sciences, Shiraz, southern Iran, 2021. A total of 534 liquid-based Papanicolaou samples were collected and transferred to the Molecular Lab of the Department of Parasitology and Mycology, Shiraz University of Medical Sciences. The demographic and clinical data of patients including age, place of residence, marital status, history of abortion, level of education, history of foamy and foul-smelling discharge, history of premature birth, history of itching of the genital area, dysuria and frequent urination, history of pain during intercourse, number of sexual partners were recorded using a questionnaire.

### DNA extraction and PCR amplification assay

The liquid-based Papanicolaou samples were centrifuged at 2500 rpm for 3 min and the pellets were used for DNA extraction using a DNA extraction kit (FavorPrep™ Blood and Tissue Genomic DNA Extraction Kit, FAVORGEN Biotech Corporation, Ping-Tung, Taiwan) according to the manufacturer’s guidelines and kept frozen at − 20 °C for further use. PCR reactions were performed in a 25 µL final volume containing 2 × red PCR premix (Taq DNA Polymerase 2x Master Mix RED, Ampliqon, Odense, Denmark), 10 pmol of each primer, and 3.5 µL of extracted DNA. A 300-base pair (bp) fragment of *T. vaginalis* genome was amplified using TVK3 (forward: 5’-ATTGTCGAACATTGGTCTTACCCTC-3′) and TVK7 (reverse: 5’-TCTGTGCCGTCTTCAAGTATGC- 3’) primers. Thermo cycler (Eppendorf 5331, Germany) program was set with an initial denaturation step at 94 °C for 5 min, then 30 cycles consisting of 90 °C for 1 min, 60 °C for 30 s, and 72 °C for 2 min, with a final extension step at 72 °C for 7 min [16]. The PCR products were analyzed by electrophoresis on 1.5% agarose gel containing gel red staining (SinaClon, Iran) and visualized under UV illumination. The reference *T. vaginalis* DNA and double distilled water were used as positive and no-template controls, respectively in each run.

### Data analysis

Statistical analysis was carried out with SPSS Software.16.0 (IBM Analytics, USA). The results were reported as mean ± standard deviation (SD). The chi-square test was used to assess whether there were significant differences between occurrences of the *T. vaginalis* DNA and variables. *P* values less than 0.05 were considered statistically significant.

## Results

In the study population, the mean age was 39.28 ± 9.89 with minimum and maximum ages of 19 and 65 years, respectively. Out of 534 participants, 494 (92.51%) were married, 7 (1.31%) were single and 33 (6.18%) were divorced. Moreover, 40 (7.49%) cases had no sexual partner and 494 (92.51%) had only one sexual partner. The majority of the women (197/534; 36.89%) had a bachelor’s degree as their highest level of education. Of 534 cases, 119 (22.28%) lived in rural areas and 415 (77.72%) lived in urban regions.

The history of abortion, history of premature birth and history of itching of the genital area, dysuria and frequent urination were reported by 62 (11.61%), 50 (9.36%), and 63 (11.79%) women, respectively. Of 534 studied women, 37 (6.93%) had foul-smelling discharge and 36 (6.74%) reported difficult or painful sexual intercourse. DNA of *T. vaginalis* was detected in 26 cases (4.86%) with a mean age of 35.23 ± 8.99 years. It was found that the age groups of 21 to 30 and 41 to 50 years have a significantly high prevalence of *T. vaginalis* infection (46.15%; *p* = 0.001 and 38.46%; *p* = 0.015, respectively). There was also a significant association between the history of foamy and foul-smelling discharge with trichomoniasis (*p* = 0.001), 22(84.62%) of the infected women had, and 4 (15.38%) did not have foamy and foul-smelling discharge. No significant association was found between other risk factors and trichomoniasis (*p* > 0.05) (Table 1).

**Table 1:** Data regarding the frequency of *Trichomonas vaginalis* in Shiraz, southern Iran

Characteristics	Overall No (%)	Positive PCR result No. (%)	*P* value
Age (Year)			
< 20	6(1.12)	0(0%)	–
21–30	101(18.91)	12(46.15%)	0.001
31–40	209(39.14)	4(15.38% (	0.782
41–50	130(24.34)	10(38.46%)	0.015
> 50	88(16.48)	0(0%)	0.99
Marital status			
Single	7(1.31)	0(0)	0.432
Married	494(92.51)	24(92.3)	
Divorced	33(6.18)	2(7.7)	
Number of sexual partners			
No sexual partner	40(7.49)	0(0%)	0.137
One sexual partner	494(92.51)	26(100%)	
Educational level			
Senior school	57(10.67)	4(15.37%)	> 0.05
High school	135(25.28)	5(19.23%)	
Associate Degree	59(11.05)	2(7.7%)	
Bachelor Graduates	197(36.89)	13(50%)	
MSc and PhD	86(16.10)	2(7.7%)	
Residence area			
Urban	415(77.72(	18(64.28)	0.67
Rural	119(22.28)	8(35.72)	
History of abortion			
Yes	62(11.61)	2(7.7%)	0.347
No	472(88.39)	24(92.3%)	
History of premature birth			
Yes	50(9.36)	1(3.85%)	0.480
No	484(90.64)	25(96.15%)	
History of itching, dysuria and frequent urination			
Yes	63(11.79)	5(19.23%)	0.376
No	471(88.21)	21(80.77%)	
History of foamy and foul-smelling discharge			
Yes	37(6.93)	22(84.62%)	0.001
No	497(93.07)	4(15.38%)	
History of pain during intercourse			
Yes	36(6.74)	3(11.54%)	0.475
No	498(93.26)	23(88.46%)	

## Discussion

Data on the detection of *T. Vaginalis* from liquid-based Papanicolaou samples of women by PCR are limited because most studies prefer to use traditional diagnostic methods [14]. The current study used liquid-based Papanicolaou samples for the detection of T. Vaginalis, and the overall rate of T. vaginalis infection was 4.86% using PCR depending on TVK3/7 gene as a target. Several studies have been performed in different regions of the world and Iran to investigate the prevalence of trichomoniasis by various methods. In a review by Arbabi et al. [17] The prevalence of *T. vaginalis* infection in the Iranian population was estimated 0.4–42%. Haghighi et al., (2019) showed that 21 (23.3%) out of 90 symptomatic women with high-risk behaviors were positive for *T. vaginalis* DNA in Zahedan, southeast Iran [18]. Their results showed that the highest prevalence in women aged 31 to 40 years may be related to high sexual activity. In another study in southwest Iran, the prevalence of *T. vaginalis* was reported 5.83%, 8.75%, and 17.5% by using wet, culture medium, and PCR techniques, respectively [15]. The higher prevalence of infection in the other studies compared to our results could be due to the target populations, in the two above studies, the women with vaginitis and high-risk behaviors were exanimated, while the current study, evaluated trichomoniasis in women who attended regular health check-ups. The type of sample collection and diagnostic techniques are also effective factors that can be other reasons for this difference.

Inconsistent with the current findings, Kim et al. [19] assessed the prevalence of trichomoniasis using PCR in vaginal discharge of 424 adult women who were referred to Gory hospital for health screening at the National Health Service, Gori, Korea. The prevalence of trichomoniasis was reported 3.3%, which was significantly higher in people over 50 years. Kriesel et al. [20] reported a 3% prevalence of *T. vaginalis* in a clinical specimen sample of 146 people in the US. However, in another study in the US by Napierala et al. [21] the prevalence of trichomoniasis in 2008 and 2010 was 8.9% and 8.6%, respectively. The prevalence in urine samples of young pregnant women was reported to be 7.7% by Miranda et al., Brazil [22]. In another study in Korea by Goo et al. [23] the prevalence of trichomoniasis was assessed using microscopic and PCR methods in vaginal swab samples. The results showed, out of 621 women 4 (0.6%) and 19 (3%) patients were positive using microscopic examination and PCR, respectively. Consistent with our results in this study the prevalence was reported less than 5% by PCR method and showed that the molecular method has a higher ability to diagnose the disease than the conventional methods. These researchers in line with our study introduced the age of 21–30 years as a high-risk age for the disease, the possibility of having high-risk sexual relations among women at this age can be a reason for it. In addition, in our study, the age group of 41–50 years was also reported as one of the high-risk ages for contracting the disease, which can be attributed to the hormonal changes in the body of women at this age, menopause, and the change in the pH of the genital area. In another study, this age range was reported as the highest prevalence of trichomoniasis in women [24].

In the current study, having foamy discharge showed a significant relationship with trichomoniasis. In a study by Arbabi et al. [25] the prevalence of *T. vaginalis* in 970 women was investigated using TYM medium and wet-mount method using vaginal discharges and urine samples. Their results showed the overall prevalence of Trichomonas infection was 2% (95% CI 2 ± 0.08). Their results showed no statistical relationship was found between clinical manifestations and parasitic results which is in contrast with the findings of the present study. Bakhtiari Nejad et al. [26] investigated the prevalence of trichomoniasis in a sample of vaginal discharge of 967 women referred to medical centers in Karaj, Iran using wet-mount, Gram staining, and culture. They showed, one (0.1%), 5 (0.5%) and 11 (1.1%) samples were positive by Gram staining, wet-mount, and Dorset culture, respectively. Their results demonstrated, a significant relationship between trichomoniasis and vaginal discharge in line with our findings.

In 2010, Depuydt et al., reported an overall prevalence of *T. vaginalis* in the general population in Flanders, Belgium of 0.37%, with the highest prevalence in women aged 41–45 years. In this study, Liquid-based cervical cytology samples from unselected women were tested by real-time quantitative PCR [24]. Junior et al., reported a 0.14% prevalence of *T. vaginalis* on Pap smear and liquid-based cytology in cervical cancer screening between 2013 and 2018 in northeastern Brazil [27].

A possible explanation for these differences in various studies might be linked to local cultures, target populations, socioeconomic statuses, personal sanitary/hygiene levels, study duration, as well as specificity and sensitivity of the detection methods. Molecular methods are providing a new procedure for detection of the parasitic infections such as *T. vaginalis* [28, 29]. PCR is one of these molecular methods which allows the amplification of one DNA molecule millions of times [30]. Traditional methods for *T. vaginalis* detection have low sensitivity from both urine samples and vaginal discharge compared with PCR [31]. Moreover, traditional methods such as culturing and staining have several disadvantages including labor-intensive, time-consuming, and loss of the most parasite characters during the fixation and staining process [32]. The Pap smear sample is a common screening method in women, while the chance of diagnosing trichomoniasis in this method is low based on pathological findings. Therefore, it is suggested that molecular methods should be used to obtain more accurate detection in liquid-based Papanicolaou samples for the diagnosis and treatment of trichomoniasis.

## Conclusion

*T. vaginalis* infection is common in liquid-based Papanicolaou samples of women who attended regular health check-ups in the study area. Screening for trichomoniasis in high-risk populations, particularly if using highly sensitive methods such as PCR, may lead to increased detection and treatment.

## Data Availability

The datasets generated during and/or analysed during the current study are available in the Figshare repository, https://figshare.com/s/6a9cc9bfc40be39c8c00.

## References

[1] World Health Organization. Global incidence and prevalence of selected curable sexually transmitted infections-2008. World Health Organization; 2012.

[2] Rowley J, Vander Hoorn S, Korenromp E, Low N, Unemo M, Abu-Raddad LJ, Chico RM, Smolak A, Newman L, Gottlieb S, Thwin SS (2019). Chlamydia, gonorrhoea, trichomoniasis and syphilis: global prevalence and incidence estimates, 2016. Bull World Health Organ.

[3] Ifeanyi OE, Chinedum OK, Chijioke UO (2018). *Trichomonas vaginalis*: complications and treatment. Int J Curr Res Med Sci.

[4] Sorvillo F, Smith L, Kerndt P, Ash L (2001). *Trichomonas vaginalis*, HIV, and African-Americans. Emerg Infect Dis.

[5] Van Gerwen OT, Camino AF, Sharma J, Kissinger PJ, Muzny CA (2021). Epidemiology, natural history, diagnosis, and treatment of *Trichomonas vaginalis* in men. Clin Infect Dis.

[6] Carrillo-Ávila JA, Serrano-García ML, Fernández-Parra J, Sorlózano-Puerto A, Navarro-Marí JM, Stensvold CR (2017). Prevalence and genetic diversity of *Trichomonas vaginalis* in the general population of Granada and co-infections with Gardnerella vaginalis and Candida species. J Med Microbiol.

[7] Ziaei Hezarjaribi H, Fakhar M, Shokri A, Hosseini Teshnizi S, Sadough A, Taghavi M (2015). *Trichomonas vaginalis* infection among iranian general population of women: a systematic review and meta-analysis. Parasitol Res.

[8] Burstein GR, Zenilman JM (1999). Nongonococcal urethritis—a new paradigm. Clin Infect Dis.

[9] Seña AC, Bachmann LH, Hobbs MM (2014). Persistent and recurrent *Trichomonas vaginalis* infections: epidemiology, treatment and management considerations. Expert Rev Anti Infect Ther.

[10] Draper D, Parker R, Patterson E, Jones W, Beutz M, French J (1993). Detection of *Trichomonas vaginalis* in pregnant women with the InPouch TV culture system. J Clin Microbiol.

[11] Lossick JG (1988). The diagnosis of vaginal trichomoniasis. JAMA.

[12] Munson KL, Napierala M, Munson E (2016). Suboptimal *Trichomonas vaginalis* Antigen Test performance in a low-prevalence sexually transmitted infection community. J Clin Microbiol.

[13] Patel SR, Wiese W, Patel SC, Ohl C, Byrd JC, Estrada CA (2000). Systematic review of diagnostic tests for vaginal trichomoniasis. Infect Dis Obstet Gynecol.

[14] Wiese W, Patel SR, Patel SC, Ohl CA, Estrada CA (2000). A meta-analysis of the Papanicolaou smear and wet mount for the diagnosis of vaginal trichomoniasis. Am J Med.

[15] Rafiei A, Safaie K, Tavalla M, Najafian M (2021). PCR detection and sequencing of *Trichomonas vaginalis* in women with suspected Vaginitis in Southwestern Iran. Infect Disord Drug Targets.

[16] Kadhim KJ, Khalaf AK. Use TVK 3/7 gene as a target to detect *Trichomonas vaginalis* from urine of women in Southern Iraq. University of Thi-Qar Journal Of Medicine. 2010, 4(1):36–46.

[17] Arbabi M, Delavari M, Fakhrieh-Kashan Z, Hooshyar H (2018). Review of *Trichomonas vaginalis* in Iran, based on Epidemiological Situation. J Reprod Infertil.

[18] Haghighi JD, Jafarimodrek M, Sohrabi S, Azizi H, Hatam-Nahavandi K (2019). Trichomoniasis prevalence at a care center amongwomen with high-risk behaviors in Zahedan, Iran. Int J High Risk Behav Addict.

[19] Kim S-R, Kim J-H, Gu N-Y, Kim Y-S, Hong Y-C, Ryu J-S (2016). Prevalence of trichomoniasis by PCR in women attending health screening in Korea. Korean J Parasitol.

[20] Kriesel JD, Bhatia AS, Barrus C, Vaughn M, Gardner J, Crisp RJ (2016). Multiplex PCR testing for nine different sexually transmitted infections. Int J STD AIDS.

[21] Napierala M, Munson E, Munson KL, Kramme T, Miller C, Burtch J, Olson R, Hryciuk JE (2011). Three-year history of transcription-mediated amplification-based *Trichomonas vaginalis* analyte-specific reagent testing in a subacute care patient population. J Clin Microbiol.

[22] Miranda AE, Pinto VM, Gaydos CA (2014). *Trichomonas vaginalis* infection among young pregnant women in Brazil. Braz J Infect Dis.

[23] Goo Y-K, Shin W-S, Yang H-W, Joo S-Y, Song S-M, Ryu J-S (2016). Prevalence of *Trichomonas vaginalis* in women visiting 2 obstetrics and gynecology clinics in Daegu, South Korea. Korean J Parasitol.

[24] Depuydt CE, Leuridan E, Van Damme P, Bogers J, Vereecken AJ, Donders GG (2010). Epidemiology of *Trichomonas vaginalis* and human papillomavirus infection detected by real-time PCR in flanders. Gynecol Obstet Invest.

[25] Arbabi M, Fakhrieh Z, Delavari M, Abdoli A (2014). Prevalence of *Trichomonas vaginalis* infection in Kashan city, Iran (2012–2013). Iran J Reprod Med.

[26] Bakhtiyar Nejad S, Fallah M, Maghsood A, Dastan D, Matini M (2018). The prevalence of Trichomoniasis in Women referring to Health Treatment Centers in Karaj City, 2016 (Iran). Qom Univ Med Sci J.

[27] Junior JE, Eleutério RMN, da Silva MNL, Marques MNA (2019). The frequency of *Trichomonas vaginalis* in pap smear and liquid-based cytology (SurePathTM) between 2013 and 2018 in a reference laboratory in Fortaleza, Brazil. J bras Doenças. Sex Transm.

[28] Lawing LF, Hedges SR, Schwebke JR (2000). Detection of trichomonosis in vaginal and urine specimens from women by culture and PCR. J Clin Microbiol.

[29] Kengne P, Veas F, Vidal N, Rey J-L (1994). Specific polymerase chain reaction diagnosis. Cell Mol Biol.

[30] Riley DE, Roberts M, Takayama T, Krieger JN. Development of a polymerase chain reaction-based diagnosis of *Trichomonas vaginalis* J Clin Microbiol. 1992, 30(2):465–472. 10.1128/jcm.30.2.465-472.1992 PMC265079 1537918

[31] Khalaf AK, Al-Nasir AHA, Al-Khayat ES. Use PCR technique to detect the infection with *Trichomonas vaginalis* among women with preterm labor. Thi-Qar Medical Journal 2016, 11(1).

[32] Petrin D, Delgaty K, Bhatt R, Garber G (1998). Clinical and microbiological aspects of *Trichomonas vaginalis*. Clin Microbiol Rev.

